# Angina Ludovici. Case presentation

**DOI:** 10.1186/1471-2334-13-S1-P42

**Published:** 2013-12-16

**Authors:** Mugurel Constantin Rusu, Sorin Hostiuc, Mihai Ceauşu, Irina Rentea

**Affiliations:** 1 Faculty of Dental Medicine, Carol Davila University of Medicine and Pharmacy, Bucharest, Romania; 2 Department of Legal Medicine and Bioethics, Carol Davila University of Medicine and Pharmacy, Bucharest, Romania; 3 Department of Forensic Pathology, National Institute of Legal Medicine, Bucharest, Romania; 4 Department of Pathology, Carol Davila University of Medicine and Pharmacy, Bucharest, Romania; 5 Department of Pathology, National Institute of Legal Medicine, Bucharest, Romania; 6 Department of Forensic Pathology, National Institute of Legal Medicine, Bucharest, Romania

## Background

Angina Ludovici (Ludwig’s angina) is a severe infection of the connective tissue from the floor of the mouth, usually occurring secondary to a tooth infection. Left untreated may cause an extrinsic obstruction of the superior respiratory tract.

## Case report

The purpose of this paper is to present an atypical case of Ludwig’s angina, whose initial presentation suggested a diagnosis of a large laryngeal tumor. The patient, 62 years old, without other known pathologies, came to the Emergency Room with severe dyspnea, dysphagia, high fever, and severe hypoxemia. It enters in respiratory arrest and dies less than 24 hours after the initial presentation. During autopsy were found signs of infection in the floor of the mouth, trachea, epiglottis, larynx, and adjacent tissues. The infective process lead to an erosion of a branch of the thyroid artery, leading to a hemorrhage in close contact with the larynx, that caused the pseudo-tumor pattern initially identified in the ER.

